# Hypoxia-Sensitive Epigenetic Regulation of an Antisense-Oriented lncRNA Controls *WT1* Expression in Myeloid Leukemia Cells

**DOI:** 10.1371/journal.pone.0119837

**Published:** 2015-03-20

**Authors:** Gregory McCarty, David M. Loeb

**Affiliations:** Department of Oncology, Division of Pediatric Oncology, Sidney Kimmel Comprehensive Cancer Center, Johns Hopkins University, Baltimore, MD, United States of America; Queen's University Belfast, UNITED KINGDOM

## Abstract

WT1 is a transcription factor expressed in hematopoietic stem cells and in most cases of myeloid leukemia. We investigated the roles of hypoxia and epigenetics in the regulation of *WT1* expression in myeloid leukemia cells. WT1 expression correlates with hypomethylation of the CpG island in Intron 1, and pharmacologic demethylation of this CpG island induces *WT1* mRNA expression. Hypoxia causes decreases in DNMT expression and activity and increased expression and activity of TET2 and TET3, resulting in demethylation of this CpG island and expression of *WT1* mRNA. Demethylation of the CpG island, either from pharmacologic treatment or induction of hypoxia, results in transcription of an antisense-oriented lncRNA, and inhibiting lncRNA expression with shRNA blocks *WT1* mRNA expression. These results reveal a novel model of hypoxia-mediated epigenetic gene regulation. In addition, this is the first report that TET2 and TET3, increasingly recognized as important epigenetic regulators of gene expression in stem cells and in cancer cells, can be regulated by hypoxia, providing a solid mechanistic link between hypoxia and epigenetic regulation of gene expression with important implications for the role of hypoxia in stem cell function.

## Introduction

WT1 is a transcription factor containing 4 zinc fingers in a C-terminal DNA binding domain [1, 2]. Although originally identified as a tumor suppressor gene in children with Wilms’ tumor, subsequent work has demonstrated that *WT1* is overexpressed in a wide variety of tumor types, including acute myeloid leukemia (AML) [3, 4]. Interestingly, expression of *WT1* is tightly regulated during development of the kidney (the organ in which Wilms’ tumor arises) and during hematopoiesis. Aberrant expression of *WT1* therefore contributes to the development of tumors arising in organs that ordinarily express *WT1* under tight developmental control. Although the prognostic significance of *WT1* expression in AML remains controversial [5–7], its importance as a tumor antigen and marker of minimal residual disease is growing [8–11]. In fact, a pilot project of the US National Cancer Institute to prioritize potential cancer vaccine antigens based on therapeutic function, immunogenicity, role in oncogenicity, specificity, expression level, number of epitopes and cellular localization listed WT1 as the top priority [12]. Given that not all leukemias express WT1, a better understanding of how WT1 expression is regulated is critical to the development of WT1-based immunotherapies.

There is increasing evidence that *WT1* expression is regulated, at least in part, by hypoxia. In a mouse model of myocardial infarction, *WT1* is upregulated in the coronary vasculature downstream of ligated coronary arteries [13], and this regulation is dependent on a hypoxia-response element (HRE) in the *WT1* promoter [14]. Our laboratory has demonstrated that *WT1* expression in sarcoma cell lines is also regulated by hypoxia, and further has shown that inhibition of this response blunts the hypoxia-mediated induction of vascular endothelial growth factor (VEGF), thus demonstrating the importance of WT1 to a normal response to hypoxia [15].

There are two CpG islands associated with the *WT1* gene locus—one at the 5’ end of the gene surrounding the promoter, and one in the first intron. Methylation of CpG islands is an important mechanism by which gene expression is regulated. CpG island methylation is the mechanism of genetic imprinting, and *WT1* has been shown to be an imprinted gene [16]. *WT1* imprinting has been implicated in renal development and disease, as well as in the development of Wilms’ tumor [17]. In previous work, we investigated whether methylation of the CpG island surrounding the *WT1* promoter influenced WT1 expression in breast cancer. Interestingly, though we found evidence of tumor-specific methylation of the promoter-associated CpG island, there was no correlation with expression in this tumor type [18].

CpG island methylation and imprinting are important for developmental gene regulation, but *WT1* is not expressed during breast development. We therefore hypothesized that despite our findings with breast cancer, CpG island methylation may play a role in regulating *WT1* expression in leukemia, since *WT1* is expressed in hematopoietic stem/progenitor cells under tight developmental control [19, 20]. We focused on the CpG island in Intron 1, which surrounds a cryptic promoter that regulates the expression of an antisense-oriented transcript which shows monoallelic expression in the developing kidney (consistent with imprinting), and this region, termed the antisense regulatory region (ARR), is hypomethylated in Wilms’ tumors with biallelic *WT1* expression [21]. We found that expression of *WT1* in both AML cell lines and in primary AML samples is tightly correlated with hypomethylation of the Intron 1 CpG island and expression of the antisense transcript, WT1 lncRNA. Demethylation of the Intron 1 CpG island with 5-azacytidine causes expression of *WT1*. Moreover, we discovered that hypoxia also leads to demethylation of the Intron 1 CpG island, expression of WT1 lncRNA, and expression of *WT1* in both AML cell lines and in primary AML samples. Induction of WT1 lncRNA is essential for hypoxia-mediated *WT1* expression, because blocking WT1 lncRNA induction with shRNA abrogates the hypoxia-mediated upregulation of *WT1*. Although there is a substantial literature investigating hypoxia-regulated histone methylation, this is the first report of a direct effect of hypoxia on cytosine methylation. Moreover, this mechanism of gene regulation, dynamic changes in CpG methylation leading to expression of a long noncoding RNA that upregulates mRNA expression, has never been described before.

## Results

### WT1 expression is correlated with hypomethylation of the Intron 1 CpG island

**Fig 1:**
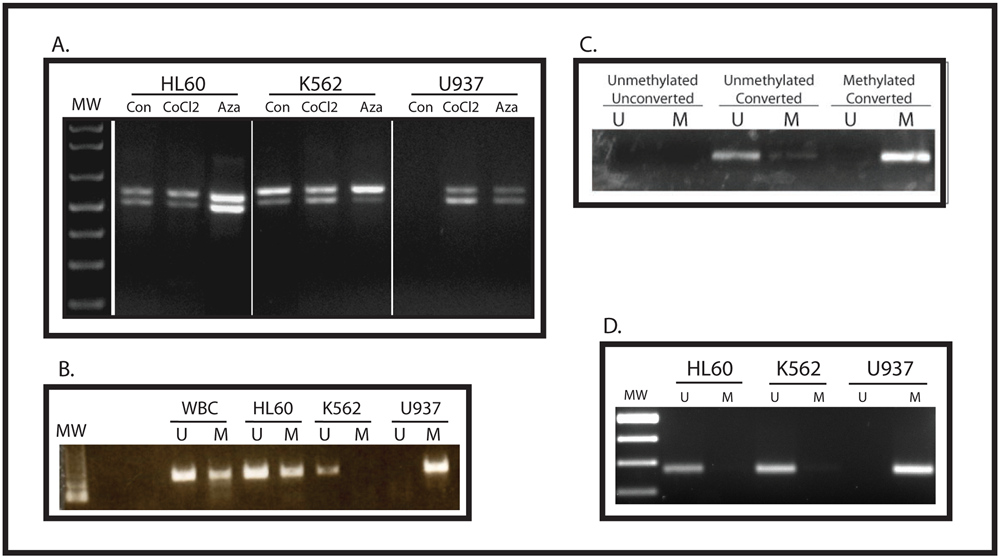
Intron 1 CpG island methylation status correlates with WT1 expression. (A) RNA was isolated from control K562, U937, and HL60 cells, as well as from cells treated with CoCl_2_ or 5-azacytidine, and was analyzed by RT-PCR using primers that span exon 5 of the WT1 mRNA. In each lane, the upper band represents the WT1 isoform containing exon 5, and the lower band represents the WT1 isoform lacking exon 5. Molecular weight markers (MW) are shown. (B) Genomic DNA was isolated from white blood cells (WBC) or from the indicated cell line, treated with sodium bisulfite, and analyzed my methylation-specific PCR using primers (validated in a previous publication [18]) specific for the WT1 promoter CpG island. U = unmethylated DNA, M = methylated DNA, MW = molecular weight markers. (C) Validation of new MSP primers. Genomic DNA was isolated from U937 cells (unmethylated) and from K562 cells (methylated) and treated with (converted) or without (unconverted) sodium bisulfite. PCR was performed with primers specific for the unmethylated, converted sequence (U) and for the methylated converted sequence (M). Each primer pair specifically amplifies the appropriate DNA sequence, and neither amplifies unmethylated unconverted DNA. (D) Genomic DNA was isolated from the indicated cell line, treated with sodium bisulfite, and analyzed by methylation-specific PCR using primers specific for the Intron 1 CpG island. U = unmethylated, M = methylated.

The *WT1* gene locus has a CpG island in the promoter region and one in Intron 1. Our previous work in breast cancer showed no correlation between methylation of the CpG island surrounding the *WT1* promoter and gene expression [18]. To investigate the relationship between methylation of the CpG island in Intron 1 and *WT1* expression, we evaluated 3 human myeloid leukemia cell lines—K562, U937, and HL60. Using RT-PCR, we confirmed that K562 and HL60 express high levels of *WT1* mRNA, but U937 cells do not (Fig. 1A). We performed methylation-specific PCR (MSP) to determine the methylation status of the promoter and of the Intron 1 CpG island. As we found in breast cancer, there was no correlation between WT1 expression and promoter methylation (Fig. 1B). Specifically, although K562 cells, which express WT1, have only unmethylated DNA, and U937 cells, which do not express WT1, have only methylated DNA, white blood cells, which do not express WT1, have both methylated and unmethylated DNA, as do HL60 cells, which do express WT1. In contrast, we found that the Intron 1 CpG island is methylated in U937 cells, but unmethylated in both K562 and HL60 (Fig. 1D). Thus, in these 3 human leukemia cell lines, the methylation status of the Intron 1 CpG island correlates with *WT1* mRNA expression.

**Fig 2:**
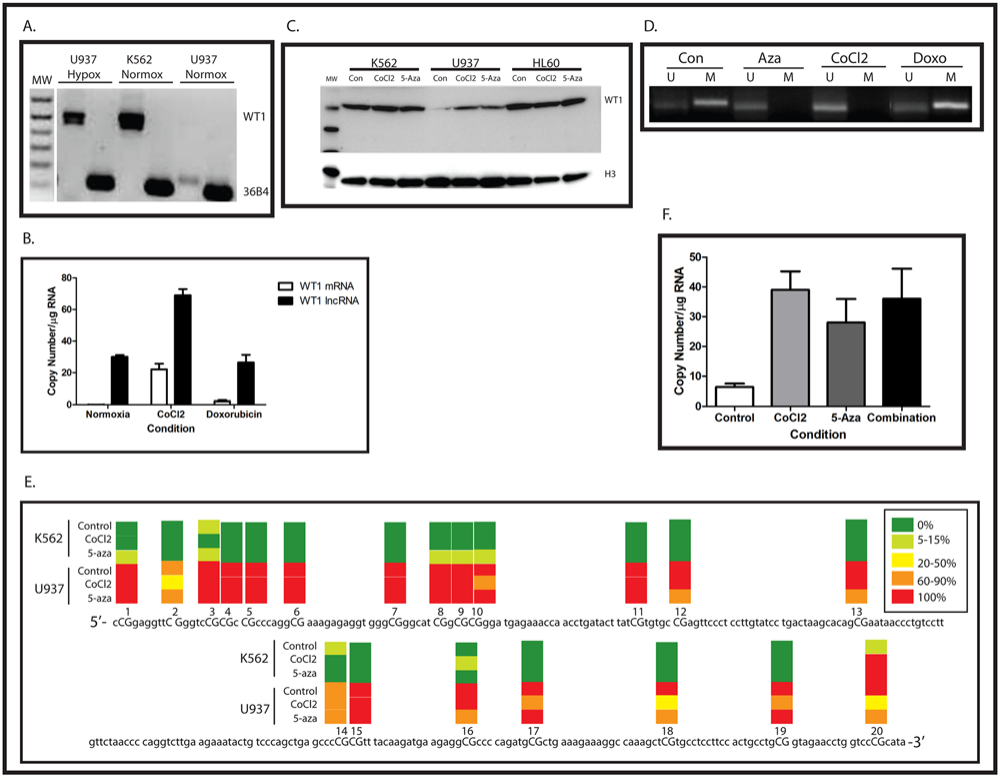
Hypomethylation of the Intron 1 CpG island results in WT1 expression. (A) RNA was isolated from K562 and U937 cells growing under atmospheric conditions (Normox) and from U937 cells growing in 1% O_2_ (Hypox) and was analyzed by RT-PCR using primers that span exon 5 of the WT1 mRNA. Ribosomal RNA 36B4 is used as a positive control, and molecular weight markers (MW) are shown. (B) U937 cells were treated with either CoCl_2_ or doxorubicin and mRNA was isolated and analyzed by quantitative RT-PCR. Treatment with CoCl_2_ led to statistically significant increases in expression of both WT1 mRNA and WT1 lncRNA significant (p<0.05 for each by Student’s t test), but treatment with doxorubicin did not. Data are presented as copy number/μg RNA, and error bars show standard error of the mean of triplicate experiments. This experiment was repeated 3 times with similar results. (C) Cell lysates from K562, U937, and HL60 cells treated with CoCl_2_, 5-azacytidine, or untreated controls were analyzed by western blotting using an antibody against WT1. Histone H3 was used as a loading control. Size of molecular weight markers is in kDa. (D) Genomic DNA was isolated from U937 cells treated with 5-azacytidine (Aza), CoCl2, or doxorubicin (doxo), as well as from untreated control cells (Con), treated with sodium bisulfite, and analyzed my methylation-specific PCR using primers specific for the Intron 1 CpG island. U = unmethylated DNA, M = methylated DNA. (E) Genomic DNA was isolated from K562 and U937 cells treated with or without CoCl_2_ or 5-azacytidine, treated with sodium bisulfite, and the CpG island in Intron 1 was sequenced. Ten independent clones from each cell line were sequenced. The genomic DNA sequence is shown, with each CpG dinucleotide capitalized and numbered. The heatmap over each CpG dinucleotide represents the degree of methylation, according to the legend. (F) U937 cells were treated overnight with either CoCl_2_, 5-azacytidine, or both, and WT1 expression was analyzed by quantitative RT-PCR. All three treatments induced similar, statistically significant (p<0.05 for each condition by Student’s t test) increases in WT1 mRNA expression. Data are presented as copy number/μg RNA, and error bars show standard error of the mean of triplicate experiments. The experiment was repeated 3 times with similar results.

Previous work from our laboratory showed that *WT1* is upregulated by hypoxia in the MHH-ES Ewing sarcoma cell line which expresses only low levels of *WT1* under normoxic conditions [15]. To determine whether WT1 is upregulated by hypoxia in leukemia cells as well, we grew U937 cells for 48 hours in 1% O_2_ and compared WT1 mRNA expression with U937 and K562 cells grown under atmospheric conditions. Similar to our published findings with Ewing sarcoma cells, hypoxia upregulates WT1 mRNA expression in U937 cells (Fig. 2A). CoCl_2_ treatment can be used to mimic hypoxia because this compound inhibits prolyl hydroxylases, stabilizing HIF-1α. We therefore treated our leukemia cell lines with 100 μM CoCl_2_ for 24 hours and collected RNA and DNA for analysis. Similar to our findings with hypoxia, CoCl_2_ strongly upregulated *WT1* mRNA expression in U937 cells (Figs. 1A and 2A). Western blotting confirmed that treatment with CoCl_2_ also upregulates WT1 protein (Fig. 2C). Thus, in AML cells, WT1 is a hypoxia-inducible gene.

Because WT1 expression under normoxic conditions is correlated with the methylation status of the Intron 1 CpG island, we investigated whether in addition to stimulating WT1 expression, CoCl_2_ could affect Intron 1 CpG island methylation. We performed MSP using the same set of primers and found that after 24 hours, CoCl_2_ induced significant hypomethylation of the Intron 1 CpG island (Fig. 2D). Because CoCl_2_ can also be a nonspecific toxin, we wanted to rule out the possibility that this compound causes hypomethylation of the Intron 1 CpG island and induction of WT1 mRNA through a nonspecific, toxic effect on U937 cells. Accordingly, cells were treated for 24 hours with 500 nM doxorubicin, and the methylation status of the Intron 1 CpG island was assessed by MSP. As expected, doxorubicin treatment did not result in significant hypomethylation of this CpG island nor did it significantly increase WT1 mRNA expression (Figs. 2B and 2D). These results suggest that the effect of CoCl_2_ on WT1 mRNA expression and on the methylation status of the CpG island is a result of stabilizing HIF-1α, rather than a nonspecific, toxic effect on the cells.

Although these results demonstrate a strong correlation between hypomethylation of the WT1 Intron 1 CpG island and mRNA expression, they do not establish a causal relationship. We therefore treated cells with the DNA methyltransferase (DNMT) inhibitor 5-azacytidine to determine if inducing hypomethylation of this region led to induction of WT1 mRNA. After 48 hours, U937 cells treated with 0.5 μM 5-azacytidine showed hypomethylation of the Intron 1 CpG island as judged by MSP (Fig. 2D) and expressed significant amounts of *WT1* mRNA (Fig. 1A) and protein (Fig. 2C).

Methylation-specific PCR is not a quantitative technique. To quantify the degree of hypomethylation induced by CoCl_2_, we confirmed the MSP results by direct sequencing of bisulfite-treated genomic DNA isolated from K562 and U937 cells. Ten independent clones from each cell line were sequenced, and using this technique we also found near-complete methylation of the Intron 1 CpG island in U937 cells, but not only minimal methylation was detected in K562 cells (Fig. 2E). In addition, direct sequencing of bisulfite-treated DNA confirmed that the Intron 1 CpG island is hypomethylated in CoCl_2_-treated U937 cells (Fig. 2E). We also confirmed our results using MSP analysis of 5-azacytidine-treated cells by sequencing bisulfite-treated genomic DNA. As expected, there was significant hypomethylation of the Intron 1 CpG island in 5-azacytidine-treated U937 cells after 48 hours (Fig. 2E). Interestingly, both CoCl_2_ and 5-azacytidine preferentially cause hypomethylation of the second CpG and of the 3’ end of the CpG island. Not surprisingly, neither CoCl_2_ nor 5-azacytidine caused complete demethylation of the CpG island in just 48 hours. The degree of demethylation observed is in line with what has been reported after 5-azacytidine treatment of a number of other CpG islands in other cell types (see, for example, [22, 23]). Thus, hypoxia causes both hypomethylation of the Intron 1 CpG island and *WT1* expression in U937 cells, and our data strongly support a causal relationship between hypomethylation of the Intron 1 CpG island and expression of *WT1* mRNA.

Because both hypoxia and hypomethylation of the Intron 1 CpG island increase WT1 expression, we investigated whether the effects of these treatments were additive. U937 cells were treated with CoCl_2_, 5-azacytidine, or both, and WT1 expression was determined using quantitative RT-PCR. Both CoCl_2_ and 5-azacytidine induced statistically significant increases in WT1 expression, and the combination increased WT1 expression by a comparable amount (Fig. 2F), supportive of a model whereby CoCl_2_ and 5-azacytidine upregulate WT1 by the same mechanism, consistent with overlapping regions of CpG hypomethylation (Fig. 2E).

### Hypoxia diminishes DNMT activity and increases TET2 and TET3

**Fig 3:**
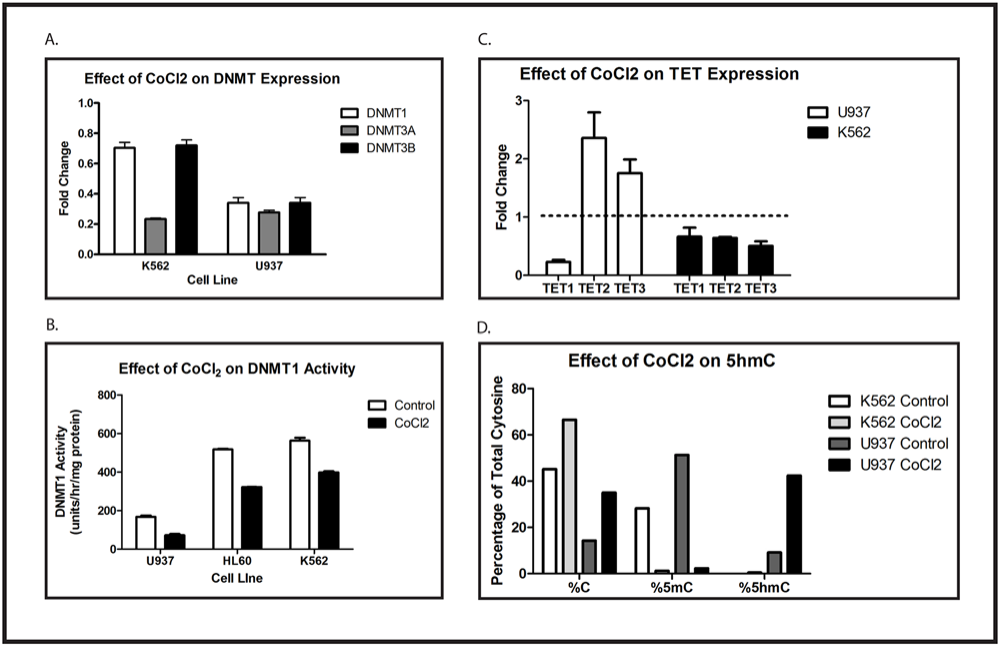
Hypoxia diminishes DNMT expression and activity and increases expression and activity of TET2 and TET3. (A) K562 and U937 cells were treated with or without 100 μM CoCl_2_ for 48 hours. Expression of DNMT1, DNMT3A and DNMT3B mRNA was evaluated by quantitative RT-PCR. Data are fold-change of CoCl_2_-treated cells compared with control. Error bar shows standard error of the mean of triplicate experiments. All changes were statistically significant, with p < 0.05. (B) U937, HL60, and K562 cells were treated overnight with or without 100 μM CoCl_2_. Protein lysates were assayed for DNMT1 activity. Error bars show standard error of the mean of triplicate experiments. Student’s t test was performed to test for statistical significance of the indicated comparisons, and all p values are < 0.0006. (C) U937 and K562 cells were treated for 48 hours with 100 μM CoCl_2_. Expression of TET1, TET2, and TET3 mRNA was evaluated by quantitative RT-PCR. Data shown are fold-change of CoCl_2_-treated cells compared with control. Error bars show standard error of the mean of triplicate experiments. Changes in U937 cells were statistically significant, with p < 0.05, but changes in K562 were not. (D) U937 and K562 cells were treated for 48 hours with 100 μM CoCl_2_. The percentage of unmodified cytosine, 5mC, and 5hmC in the WT1 locus were quantified as described. Data shown are the means of triplicate samples, and this experiment was repeated 3 times with similar results. No statistical analysis of the data presented in Fig. 2D was performed because the number of transformations applied to the raw data in order to calculate the presented results (see Materials and Methods) makes all standard parametric tests of significance meaningless. Nevertheless, one can calculate that the likelihood of the observed pattern (comparing control and CoCl_2_-treated samples in each cell line) occurring by chance in a single experiment is 1/2^6^, or 1.5%, and the likelihood of seeing this pattern in 3 independent experiments is 0.015^3^, or 3.4 x 10^-6^. Each experiment in this figure was repeated at least 3 times with similar results.

The decrease in methylation of the Intron 1 CpG island caused by hypoxia could result from either active DNA demethylation, a reduction in activity of the DNMTs that maintain the methylation status, or both. Several DNMTs have been implicated in establishing and maintaining DNA methylation patterns. We used quantitative RT-PCR to determine whether CoCl_2_ treatment affects the expression of any of these enzymes. K562 and U937 cells were treated for 48 hours with or without 100 μM CoCl_2_. We found that this treatment causes a significant decrease in DNMT3A expression in K562 cells, as well as modest decreases in expression of DNMT1 and DNMT3B (Fig. 3A). In U937 cells, CoCl_2_ causes substantial decreases in the expression of all of these enzymes (Fig. 3A). Because DNMT1 has been implicated in the maintenance of established CpG island methylation patterns, we investigated the effect of CoCl_2_ on DNMT1 activity in these cell lines. Using an *in vitro* assay, we found that CoCl_2_ induces modest, reproducible decreases in DNMT1 activity in K562 and HL60 cells (treated k562 cells have 70.1% of control activity, and treated HL60 cells have 62% of control activity; Fig. 3B), whereas treated U937 cells have only 43% of control activity, and this is the line in which CoCl_2_ induces hypomethylation of the Intron 1 CpG island.

In addition to inhibition of DNMT activity, CoCl_2_ might also influence CpG island methylation by inducing active DNA demethylation. Recently, the Ten-Eleven-Translocation (TET) proteins have attracted significant attention because of their ability to reverse cytosine methylation by catalyzing the oxidation of 5-methylcytosine (5mC) to 5-hydroxymethylcytosine (5hmC), which is subsequently converted to unmodified cytosine. This has been postulated to be the primary mechanism by which 5mC is removed from genomic DNA. We therefore investigated whether CoCl_2_ modulates TET expression in K562 and U937 cells. Cells were treated for 48 hours with or without 100 μM CoCl_2_ and expression of TET1, TET2, and TET3 was quantified using RT-PCR. Although we saw only minimal changes in TET mRNA expression in K562 cells, CoCl_2_ caused approximately 2-fold increases in both TET2 and TET3 in U937 cells (Fig. 3C). Interestingly, CoCl_2_ also caused an 80% decrease in TET1 expression in U937 cells, but not in K562 cells. To confirm the functional importance of the CoCl_2_-mediated upregulation of TET2 and TET3, we determined the relative expression of 5hmC in K562 and U937 cells treated with or without CoCl_2_. As anticipated, control K562 cells had more unmodified cytosine than 5mC at the *WT1* locus, whereas in untreated U937 cells there was a preponderance of 5mC (Fig. 3D). As would be expected from our TET RT-PCR results, CoCl_2_ had no significant effect on 5hmC at the *WT1* locus in K562 cells. In contrast, CoCl_2_ induced a marked increase in 5hmC at the *WT1* locus in U937 cells (Fig. 3D), consistent with increased TET enzymatic activity. Thus, the CoCl_2_-mediated loss of *WT1* intron 1 CpG island methylation probably results from a combination of passive demethylation (decreased DNMT1 activity) and active demethylation (increased expression and activity of TET2 and TET3).

### Expression of an antisense-oriented long non-coding RNA is under epigenetic regulation and correlates with WT1 expression

**Fig 4:**
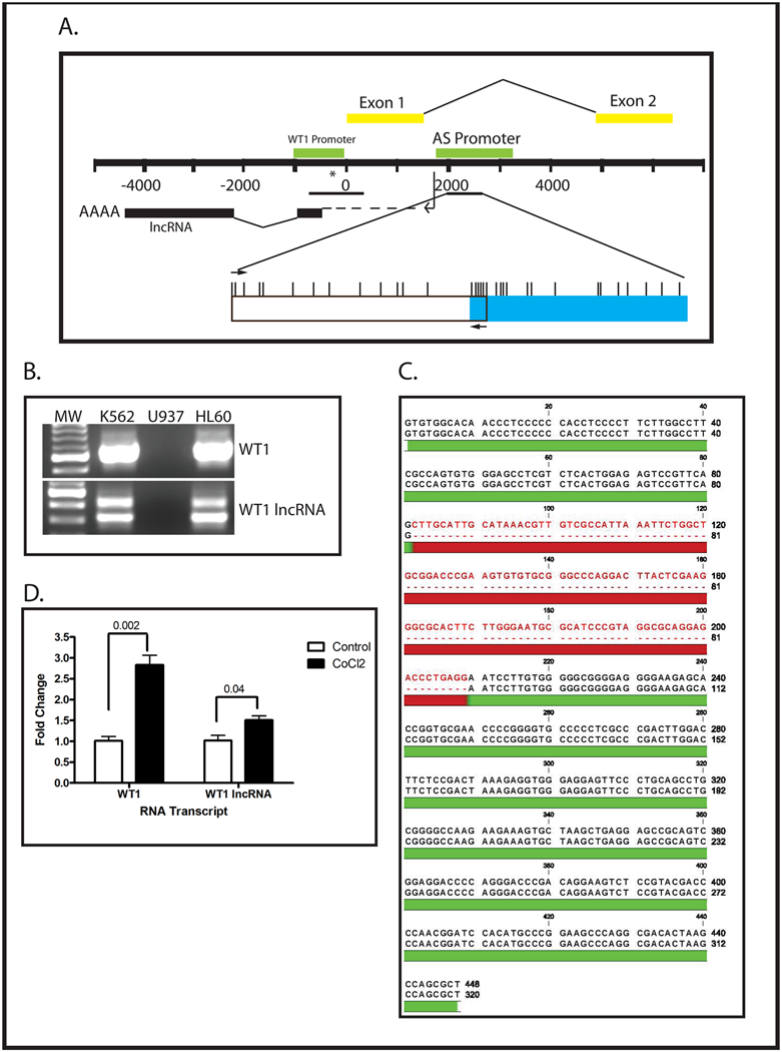
WT1 mRNA and WT1 lncRNA are co-expressed from the same locus. (A) Schematic of the *WT1* gene locus. Numbers represent position relative to the *WT1* mRNA transcription start site. The yellow bars show exons 1 and 2 of *WT1* mRNA. The antisense-oriented lncRNA promoter (AS Promoter) is the green bar in intron 1 of *WT1*, and the position of the transcribed lncRNA is indicated by the black bar. The lncRNA transcription start site is located ~200 bp upstream of the *WT1* mRNA transcription start site. The position of the promoter CpG island and Intron 1 CpG island are indicated by the horizontal black lines. The position of the HRE is indicated by (*). A detailed view of the Intron 1 CpG island, including locations of each CpG (vertical hash marks) and the primers used for MSP (arrows) is provided below. The blue box indicates the regions that was subjected to bisulfite sequencing in Fig. 2E. (B) RNA was isolated from K562, U937, and HL60 cells and subjected to RT-PCR with primers specific for *WT1* mRNA (top panel) or WT1 lncRNA (lower panel). Note that the *WT1* mRNA primers are different from the ones used in Fig. 1, do not span an alternatively spliced region, and only amplify a single band. (C) Sequence of the bands amplified by the WT1 lncRNA primers used in panel B. The region in red is present in the larger isoform and absent from the smaller isoform. (D) K562 cells were treated with or without 100 μM CoCl_2_ overnight and analyzed by quantitative RT-PCR using primers specific for either *WT1* mRNA or WT1 lncRNA. Data presented are fold change relative to untreated (control), and error bars show standard error of the mean of triplicate assays. Both transcripts show statistically significant increases in expression, as judged by Student’s t test, with the indicated p value. Experiments were repeated 3 times with similar results.

The ARR, in Intron 1, has been hypothesized to contain a cryptic promoter that controls the expression of an RNA encoded by the antisense strand of the *WT1* gene (Fig. 4A), and expression of that noncoding RNA has been correlated with *WT1* mRNA expression in developing kidney [24]. We therefore investigated whether expression of WT1 lncRNA (the antisense-oriented noncoding RNA) correlates with the methylation status of the Intron 1 CpG island in human leukemia cells. We designed PCR primers specific for the WT1 lncRNA (Table 1) and performed qualitative RT-PCR using RNA isolated from K562, U937 and HL60 cells grown under normoxic conditions. We found that 2 bands were amplified from K562 and HL60 cells, but none from U937 (Fig. 4B). These bands were individually purified and sequenced (Fig. 4C). This analysis demonstrated that in K562 and HL60 cells there are 2 isoforms of the WT1 lncRNA which appear to result from alternative splicing of the antisense-oriented transcript. Next, we isolated total RNA from U937 and K562 cells grown with or without 100 μM CoCl_2_ for 48 hours and determined WT1 mRNA and WT1 lncRNA expression using quantitative RT-PCR. As reflected in our qualitative RT-PCR data (Fig. 1A), K562 cells express a substantial amount of *WT1* mRNA (134,552 ± 5480 copies per μg RNA), while the *WT1*-nonexpressing U937 cells express almost none (15 ± 5 copies per μg RNA). We also confirmed our qualitative RT-PCR findings (Fig. 4B) that normoxic K562 cells express substantial WT1 lncRNA (92,288 ± 4527 copies per μg RNA), while U937 cells express almost none (11 ± 6 copies per μg RNA). Treatment with CoCl_2_, which results in hypomethylation of the Intron 1 CpG island in U937 cells (Figs. 2D and E) resulted in measureable expression of *WT1* mRNA (8513 ± 7014 copies per μg RNA) and WT1 lncRNA (294 ± 88 copies per μg RNA). K562 cells, which already express both *WT1* and WT1 lncRNA, showed a modest 50% increase in WT1 lncRNA expression and 2.8-fold increase in *WT1* mRNA expression (Fig. 4D). Thus, CoCl_2_ causes hypomethylation of the *WT1* Intron 1 CpG island, resulting in increased expression of both *WT1* mRNA and WT1 lncRNA. To rule out the possibility that induction of both WT1 lncRNA and *WT1* results from nonspecific toxicity related to treatment with CoCl_2_, we treated U937 cells with 500 nM doxorubicin for 24 hours, isolated RNA, and performed quantitative RT-PCR. Neither *WT1* mRNA nor WT1 lncRNA was upregulated by doxorubicin (Fig. 2B). Thus, the induction of WT1 lncRNA and WT1 mRNA by CoCl_2_ in these cells is a specific effect, most likely related to stabilization of HIF-1, rather than reflecting a nonspecific cellular response to cytotoxicity.

**Table 1:** Primer Sequences.

Primer	Target	Sequence
**376**	WT1 int1 ChiP qPCR f	CGAAGGTACCTCCTGCAAAA
**375**	WT1 int1 ChiP qPCR r	CCCCTGTAGTTTGCCCTCTT
**220**	WT1/WT1 lncRNA TSS ChIP qPCR f	AACCCAACACGCGCTCTCA
**339**	WT1/WT1 lncRNA TSS ChIP qPCR r	TCTCTACTCCCACCGCATTC
**225**	WT1 lncRNA f	GTGTGGCACAACCCTCCCCC
**226**	WT1 lncRNA r	AGCGCTGGCTTAGTGTCGCC
**3946**	WT1 ex5 f	GCGGCGCAGTTCCCCAACCA
**3945**	WT1 ex5 r	ATGGTTTCTCACCAGTGTGCTT
**36B4s**	36B4 PCR Control f	GATTGGCTACCCAACTGTTGCA
**36B4a**	36B4 PCR Control r	CAGGGGCAGCAGCCACAAAGGC
**1Uf**	WT1int1 MSP unmethylated f	GTGTGGGTGAAGGTGGGTAAT
**1Ur**	WT1int1 MSP unmethylated r	CAAACCCAAACCTACAAAACC
**1Mf**	WT1int1 MSP methylated f	GCGCGGGTGAAGGCGGGTAAT
**1Mr**	WT1int1 MSP methylated r	CGAACCCGAACCTACGAAACC
**161**	WT1int1 bis seq f	TGTTTTTGGTTAGGTTAAGGTA
**166**	WT1int1 bis seqr	ACCTCCAATAACTAAATAACTTTC
**388**	5-hmC qPCR f	TTAACAAAACTCTCCCCAAGG
**389**	5-hmC qPCR r	AGCCAGGCAGAGCTAGGAG

**Fig 5:**
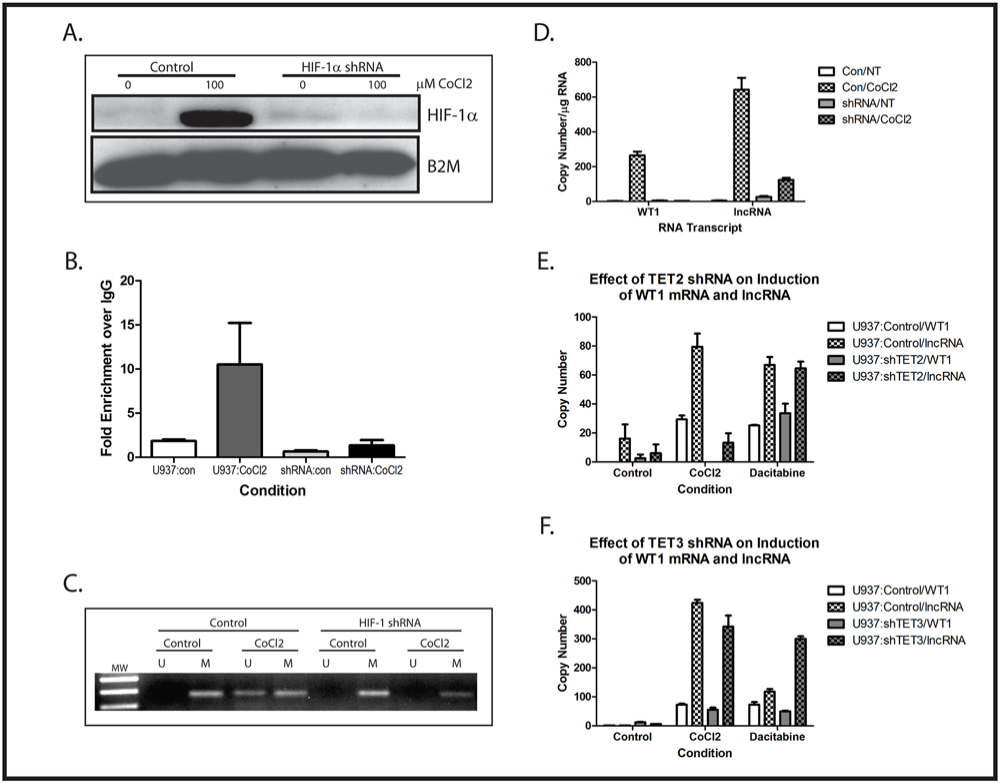
Importance of HIF-1α and TET2/3 in CoCl_2_-mediated induction of WT1 and WT1 lncRNA. (A) U937 cells stably transfected with either HIF-1α shRNA or a scramble control were treated with or without 100 μM CoCl_2_ overnight. Total cellular protein was isolated and analyzed by western blotting with anti-HIF-1α antibody. Stabilization of HIF-1α is seen in CoCl_2_-treated cells, but this is inhibited by the HIF-1α shRNA. This and the other experiments in this figure were repeated with a second shRNA with similar results. (B) Chromatin immunoprecipitation assays, with quantitative PCR, were performed using antibodies specific for HIF-1α to evaluate binding to the HRE in the WT1 promoter in U937 cells transfected with either the HIF-1α shRNA or a scramble control. Cells were treated with or without 100 μM CoCl_2_ overnight, and chromatin was isolated, immunoprecipitated with anti-HIF-1α antibody or an isotype control, and analyzed by quantitative PCR using primers surrounding the WT1 HRE. Fold enrichment compared with isotype control was calculated. Data are fold change relative to untreated (control), and error bars show standard error of the mean of triplicate assays. CoCl_2_ induced a statistically significant (p<0.05) increase in HIF-1α binding to the HRE in control cells, but not in cells stably transfected with HIF-1α shRNA. (C) U937 cells stably transfected with HIF-1α shRNA or a scramble control were treated with or without (control) 100 μM CoCl_2_ overnight. Genomic DNA was isolated, treated with sodium bisulfite, and the methylation status of the intron 1 CpG island was assessed by methylation-specific PCR. Treatment with CoCl_2_ caused demethylation of the CpG island in control cells, but not in cells expressing the HIF-1α shRNA. (D) U937 cells transfected with HIF-1α shRNA (shRNA) or a scramble control (Con) were treated with or without (NT = no treatment) 100 μM CoCl_2_ overnight and analyzed by quantitative RT-PCR using primers specific for WT1 or the WT1 lncRNA. Data are copy number per μg RNA, and error bars show standard error of the mean of triplicate assays. (E) U937 cells stably transfected with TET2 shRNA or scramble control were treated for 48 hours with either 100 μM CoCl2 or 0.3 μM dacitabine and analyzed by quantitative RT-PCR for WT1 and lncRNA expression. Data are shown as copy number per μg RNA, and error bars represent standard error of the mean of triplicate experiments. Statistical analysis using Student’s t test showed significant (p<0.05) increases in both WT1 and lncRNA mediated by dacitabine in both cell lines, but only by CoCl_2_ in the TET2 knock down cell line. (F) U937 cells stably transfected with TET3 shRNA or scramble control were treated for 48 hours with either 100 μM CoCl2 or 0.3 μM dacitabine and analyzed by quantitative RT-PCR for WT1 and lncRNA expression. Data are shown as copy number per μg RNA, and error bars represent standard error of the mean of triplicate experiments. Statistical analysis using Student’s t test showed significant (p<0.05) increases in both WT1 and lncRNA mediated by both CoCl_2_ and dacitabine. All experiments were repeated a minimum of 3 times with similar results.

To confirm the importance of HIF-1 in controlling the hypomethylation of the Intron 1 CpG island and expression of WT1 mRNA and WT1 lncRNA, and further support our contention that CoCl_2_ upregulates WT1 through stabilization of HIF-1α, U937 cells were stably transduced with HIF-1α-specific shRNA. Two independent clones, as well as a clone expressing a scramble shRNA, were selected for further evaluation. These cells were treated with or without 100 μM CoCl_2_ for 48 hours. Western blotting showed stabilization of HIF-1α protein by CoCl_2_, which was not seen in cells stably expressing HIF-1α shRNA (Fig. 5A). Chromatin immunoprecipitation experiments demonstrated that HIF-1α binds to the HRE located in the WT1 promoter region when U937 cells are treated with CoCl_2_ (Fig. 5B). Genomic DNA was isolated and the methylation status of the Intron 1 CpG island was evaluated by MSP. Although the scramble shRNA had no effect on the ability of CoCl_2_ to induce hypomethylation of the Intron 1 CpG island, this change was blocked by the HIF-1α shRNA (Fig. 5C). We also measured induction of WT1 mRNA and of WT1 lncRNA in the U937 cells stably expressing HIF-1α shRNA. Cells were treated with 100 μM CoCl_2_ for 48 hours and analyzed by quantitative RT-PCR using primers specific for either WT1 mRNA or the WT1 lncRNA. As expected, the scramble shRNA had no impact on the induction of WT1 lncRNA and WT1 mRNA expression by CoCl_2_. In contrast, the HIF-1α shRNA abolished induction of both RNAs by CoCl_2_ (Fig. 5D). These data confirm that the ability of CoCl_2_ to modulate both methylation of the Intron 1 CpG island and expression of WT1 and WT1 lncRNA is dependent on HIF-1α.

To confirm the importance of active demethylation mediated by increased TET activity in the regulation of *WT1* mRNA and lncRNA expression, and to investigate the relative importance of TET2 compared to TET3, we generated U937 cell lines lacking either TET2 or TET3 expression by stably transfecting with the respective shRNAs. Two independent clones of U937 expressing either TET2 or TET3 shRNA were isolated and knockdown confirmed by RT-PCR (data not shown). U937 cells deficient in TET2 upregulated neither *WT1* nor WT1 lncRNA in response to 100 μM CoCl_2_ (Fig. 5E). As would be anticipated, bypassing active demethylation mediated by TET2 by treating cells with 0.3 μM decitabine, a DNMT inhibitor, resulted in strong upregulation of both *WT1* mRNA and WT1 lncRNA (Fig. 5E). In stark contrast, knocking down TET3 expression had no effect on induction of *WT1* mRNA or lncRNA in U937 cells by CoCl_2_ (Fig. 5F). These results clearly demonstrate that TET2 is the enzyme that mediates CoCl_2_-induced demethylation of the CpG island in intron 1, leading to upregulation of the lncRNA and of the mRNA and confirm the importance of this demethylation event by demonstrating that loss of TET2 can be bypassed by inducing passive demethylation with decitabine.

**Fig 6:**
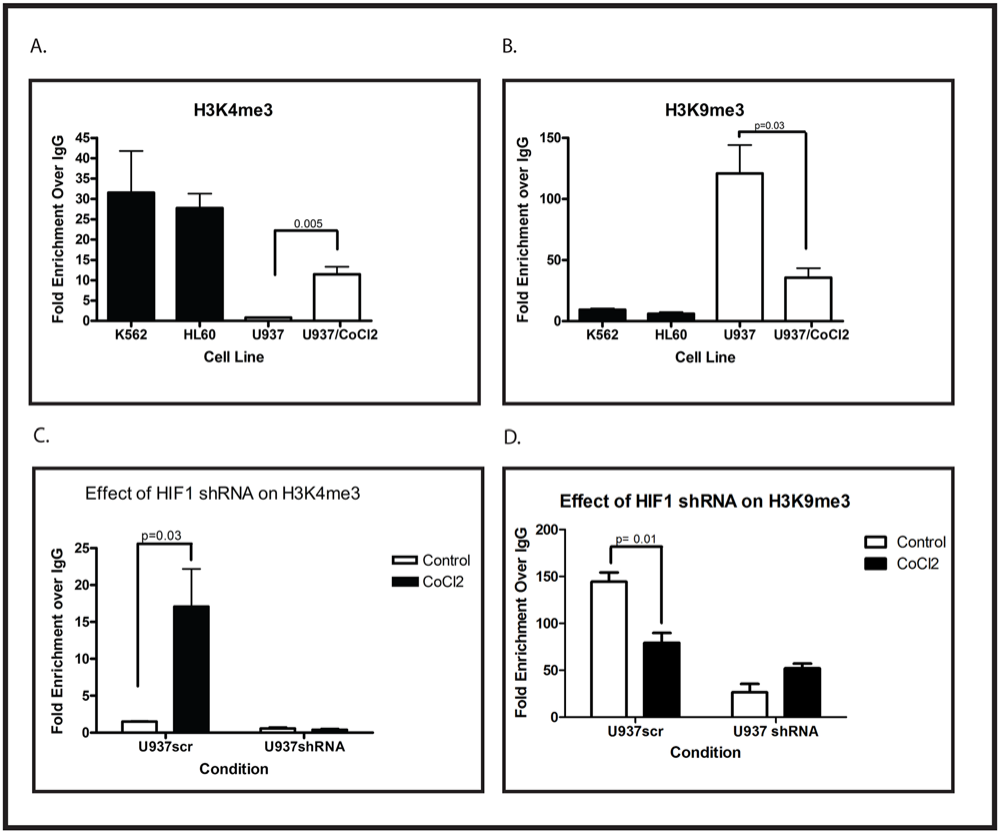
CoCl_2_-mediated changes in histone methylation around the WT1 transcription start site. (A and B) Chromatin immunoprecipitation assays, with quantitative PCR, were performed using antibodies specific for H3K4me3 (A) or H3K9me3 (B) on material isolated from K562 cells, HL60 cells, or U937 cells with or without 100 μM CoCl_2_ treatment for 48 hours. Error bars show standard error of the mean for triplicate assays. The changes induced by CoCl_2_ were confirmed to be statistically significant (p values shown) by Student’s t test. (C and D) U937 cells stably transfected with an shRNA specific for HIF-1α or a scramble control (scr) were treated with or without 100 μM CoCl_2_ for 48 hours and analyzed by chromatin immunoprecipitation, with quantitative PCR, using antibodies specific for H3K4me3 (C) or H3K9me3 (D). Statistically significant differences (Student’s t test p value shown) were seen in control (scr) cells but not in cells expressing the shRNA. Error bars show standard error of the mean for triplicate assays. All experiments were repeated at least 3 times with similar results.

In addition to cytosine methylation, histone methylation is an important epigenetic regulator of transcription. There are several lysine residues on histone H3 that can be methylated, and although the significance of some of these modifications remains unclear, it is reasonably well established that methylation of lysine 4 (H3K4me) marks activated genes, and methylation of lysine 9 (H3K9me) is associated with transcriptional silencing. We used chromatin immunoprecipitation to investigate the methylation status of H3K4 and H3K9 surrounding the WT1 lncRNA transcription start site in U937, HL60, and K562 cells. Under normoxic conditions in K562 and HL60 cells, this region is characterized by the presence of substantial amounts of trimethylated H3K4 and very little trimethylated H3K9, consistent with active transcription (Figs. 6A and B). In contrast, in U937 cells, this region contains significant trimethylated H3K9 and little trimethylated H3K4 (Figs. 6A and B). Treating U937 cells with 100 μM CoCl_2_ results in the induction of trimethylated H3K4 and the suppression of trimethylated H3K9 (Figs. 6A and B), consistent with activation of WT1 lncRNA transcription, leading to transcription of *WT1*. Induction of trimethylated H3K4 by CoCl_2_ is blocked by the HIF-1α shRNA (Fig. 6C), as is suppression of trimethylated H3K9 (Fig. 6D), consistent with a direct role of HIF-1 in causing these changes.

### WT1 lncRNA regulates WT1 mRNA expression

**Fig 7:**
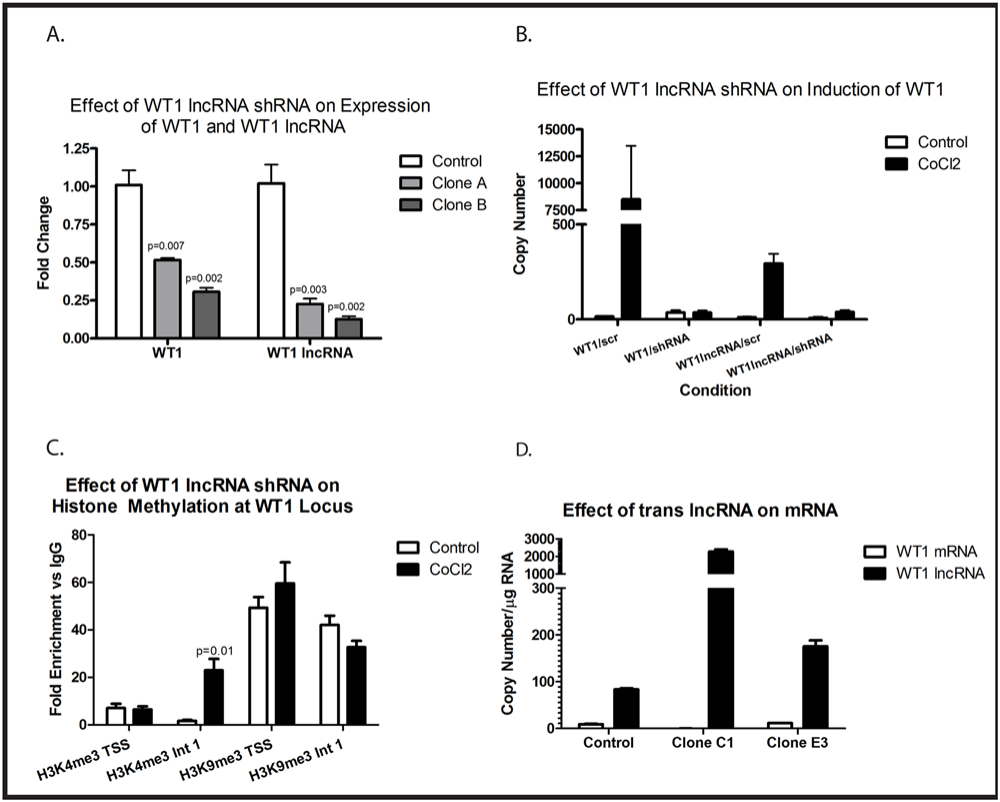
WT1 lncRNA is necessary for WT1 expression and changes in histone methylation but does not induce WT1 expression in trans. (A) K562 cells stably transfected with either a scramble shRNA (control) or an shRNA specific for the lncRNA (clones A and B) were evaluated by quantitative RT-PCR for expression of WT1 and WT1 lncRNA. Bars show fold change in expression relative to control. Error bars show standard error of the mean for triplicate assays. The reduction of WT1 and WT1 lncRNA expression in each clone was statistically significant (Student’s t test, p values shown). (B) U937 cells stably transfected with either the WT1 lncRNA shRNA or a scramble control (scr) were treated with or without 100 μM CoCl_2_ for 48 hours. Expression of *WT1* and WT1 lncRNA were assessed by quantitative RT-PCR. Bars show copy number per μg RNA, and error bars show standard error of the mean for triplicate assays. (C) Chromatin immunoprecipitation was performed on material isolated from U937 cells stably expressing WT1 lncRNA shRNA treated with or without (control) 100 μM CoCl_2_ for 48 hours using antibodies specific for H3K4me3 and H3K9me3 and primers specific for the *WT1* transcription start site (TSS) or intron 1 (int 1). Bars show fold enrichment compared with nonspecific IgG, and error bars represent standard error of the mean of triplicate assays. Only the induction of H3K4me3 in intron 1 reached statistical significance, as judged by Student’s t test (p value shown). (D) U937 cells stably transfected with WT1 lncRNA-A (Clone C1 and Clone E3) or an empty expression vector (Control) were evaluated by quantitative RT-PCR for expression of WT1 and WT1 lncRNA. Bars show copy number per μg RNA, and error bars show standard error of the mean for triplicate assays. Experiments were repeated a minimum of 3 times with similar results.

We next investigated the possibility of a causal relationship between WT1 lncRNA expression and expression of *WT1* mRNA. We used shRNA that targets both isoforms of WT1 lncRNA to determine if interfering with expression can affect *WT1* mRNA expression. K562 cells, which express high levels of *WT1*, were transduced with a plasmid directing expression of either the WT1 lncRNA shRNA or a scramble control. Two independent clones expressing the shRNA were isolated and evaluated. Compared with a K562 clone stably expressing the scramble shRNA, both clones expressing the shRNA directed against WT1 lncRNA contained significantly less WT1 lncRNA and *WT1* mRNA (Fig. 7A). We next wished to determine if upregulation of WT1 lncRNA by hypoxia was necessary for upregulation of *WT1* mRNA. U937 cells were therefore transduced with plasmids directing expression of either the WT1 lncRNA shRNA or the scramble control. The WT1 lncRNA shRNAs both effectively abolished the CoCl_2_-mediated upregulation of both WT1 lncRNA and *WT1* mRNA, while the scramble shRNA had no effect (Fig. 7B). Because the mRNA and lncRNA share no sequences in common, and thus the shRNA targeting the lncRNA cannot be directly affecting mRNA stability (confirmed by BLAST analysis of the shRNA), these findings demonstrate that expression of WT1 lncRNA appears to be necessary for both baseline expression of *WT1* mRNA in K562 cells and for induction of *WT1* mRNA in U937 cells by hypoxia.

There is increasing evidence suggesting that lncRNAs regulate mRNA expression through epigenetic mechanisms. Our data demonstrate a correlation between the histone methylation status of the *WT1* transcription start site and *WT1* mRNA expression. Because WT1 lncRNA is necessary for *WT1* mRNA expression, we investigated whether WT1 lncRNA modulates histone methylation at the *WT1* mRNA transcription start site. U937 cells stably transfected with shRNA specific for the WT1 lncRNA were treated with or without 100 μM CoCl_2_, and H3K4 and H3K9 methylation were assessed by chromatin immunoprecipitation using PCR primers specific to either the WT1 mRNA transcription start site or to Intron 1. The WT1 lncRNA shRNA blocked the CoCl_2_-mediated increase in H3K4me3 and CoCl_2_-mediated decrease in H3K9me3 previously seen at the WT1 transcription start site (Fig. 7C). Interestingly, CoCl_2_ still upregulated H3K4me3 in intron 1, surrounding the WT1 lncRNA promoter. Downregulation of H3K9me3 in Intron 1 was modest in the presence of the shRNA (Fig. 7C). Thus, the CoCl_2_-mediated epigenetic changes associated with increased *WT1* mRNA transcription require induction of the WT1 lncRNA.

To begin to address whether expression of WT1 lncRNA is not only necessary, but also sufficient to induce expression of *WT1* mRNA, we cloned the WT1 lncRNA-A isoform into a retroviral expression vector and stably transduced these vectors into U937 cells. Two independent clones that stably overexpress WT1 lncRNA-A were identified. Expression of exogenous WT1 lncRNA was confirmed by quantitative RT-PCR, but neither of the clones express significant amounts of *WT1* mRNA (Fig. 7D) suggesting that expression of WT1 lncRNA in trans is insufficient to induce *WT1* expression in this cell line.

### WT1 expression correlates with hypomethylation of the Intron 1 CpG island in primary leukemia samples

**Fig 8:**
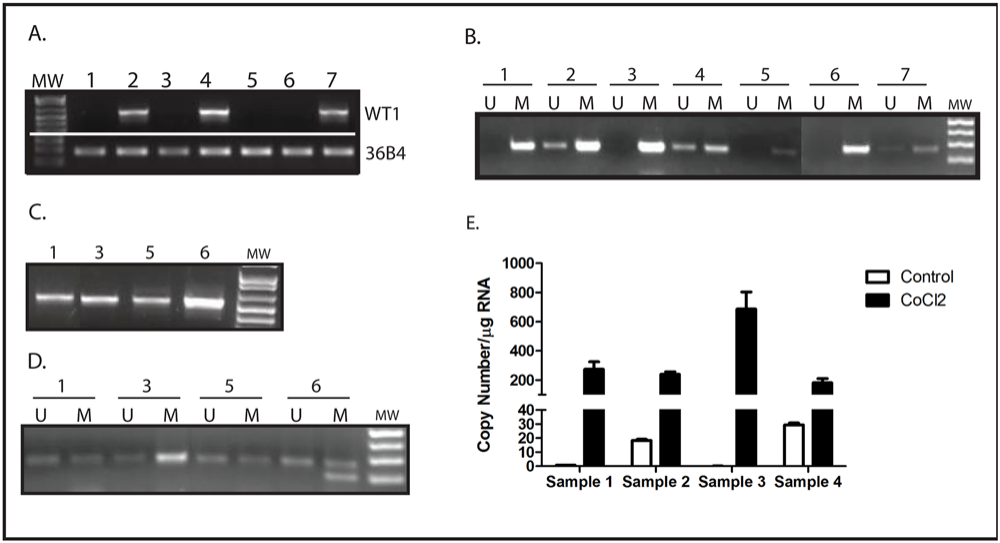
CoCl_2_ induces CpG island demethylation and WT1 expression in primary AML samples. (A) Expression of WT1 in 7 independent primary AML samples was evaluated by RT-PCR. The ribosomal RNA 36B4 was used as a loading control. (B) Genomic DNA from the same AML samples was isolated, treated with sodium bisulfite, and evaluated by methylation-specific PCR using primers specific for WT1 intron 1. Unmethylated DNA (U) was only detected in the samples with WT1 expression, and methylated DNA (M) was detected in the samples without WT1. (C) The 4 WT1-negative samples were cultured for 48 hours with 100 μM CoCl_2_ and WT1 expression analyzed by RT-PCR. (D) Genomic DNA was isolated from the 4 WT1-negative samples after treatment with 100 μM CoCl_2_. DNA was treated with sodium bisulfite and the methylation status (unmethylated = U, methylated = M) of the intron 1 CpG island was determined by methylation-specific PCR. (E) Expression of WT1 lncRNA was analyzed by quantitative RT-PCR in 4 of the primary AML samples before and after treatment with 100 μM CoCl_2_. Bars show copy number per μg RNA, and error bars show standard error of the mean for triplicate assays.

To ensure that the phenomenon of hypoxia-mediated hypomethylation of the *WT1* Intron 1 CpG island resulting in upregulation of both WT1 lncRNA and *WT1* mRNA is not an artifact of cell culture, we evaluated Intron 1 methylation status and expression of both *WT1* mRNA and WT1 lncRNA in a panel of primary acute myeloid leukemia (AML) samples. Using standard RT-PCR, we screened a panel of AML samples for *WT1* expression and identified 3 that were *WT1*-positive and 4 that were *WT1*-negative (Fig. 8A). Using MSP, we found a perfect correlation between *WT1* expression and hypomethylation of the Intron 1 CpG island (Fig. 8B). As was seen with the cell lines, CoCl_2_ treatment upregulates *WT1* expression (Fig. 8C) and causes hypomethylation of the Intron 1 CpG island (Fig. 8D) in the *WT1*-negative AML samples. In the 4 samples with sufficient material available for analysis, WT1 lncRNA expression was investigated using RT-PCR. Samples 2 and 4, which express WT1 mRNA (Fig. 7A), express measurable amounts of WT1 lncRNA (Fig. 8E), while samples 1 and 3, which do not express WT1 mRNA (Fig. 7A), also do not express WT1 lncRNA (Fig. 8E). Importantly, WT1 lncRNA expression is upregulated by CoCl_2_ in all 4 samples (Fig. 8E). Thus, the novel mechanism of regulation of WT1 expression uncovered in myeloid leukemia cell lines appears to be functional in primary AML samples as well.

## Discussion

WT1 is a transcription factor that plays key roles in both embryogenesis and tumorigenesis. Despite tight developmental regulation and playing a critical role in major biological processes, very little is understood regarding how WT1 expression is regulated. In 1994, Jinno et al. reported that although WT1 is biallelically expressed in the kidney, in 5 of 9 preterm placentae WT1 was expressed largely from the maternal allele, and monoallelic expression was also found in 2 fetal brains, suggesting that WT1 can undergo tissue-specific imprinting [16]. Because DNA methylation plays a prominent role in this process [25], and in regulating tumor-specific silencing of tumor suppressor genes [26], we have previously investigated whether the methylation status of the CpG island surrounding the WT1 promoter influences expression in breast cancer. Interestingly, although we found evidence for tumor-specific methylation of this CpG island, we found no correlation with expression [18]. In contrast, in the present study we found a perfect correlation between methylation of the Intron 1 CpG island and silencing of WT1, in both cell lines and in primary AML samples. Thus, it appears that a major regulator of WT1 expression, at least in leukemia, is the methylation status of the CpG island in Intron 1.

Recent evidence suggests that WT1 expression is also regulated by hypoxia. Wagner et al. have demonstrated that upregulation of WT1 in endothelial cells after coronary artery ligation is dependent on a hypoxia-response element in the promoter [13, 14], and our laboratory showed that, in Ewing sarcoma cell lines, upregulation of WT1 by hypoxia results in increased expression of VEGF [15]. We therefore investigated whether upregulation of WT1 by hypoxia is accompanied by a change in methylation of the Intron 1 CpG island. Indeed, we found that hypoxia leads to hypomethylation of the Intron 1 CpG island, which leads to expression of WT1 mRNA and protein. Although most of our experiments were performed using CoCl_2_, we provide substantial evidence that the effect of CoCl_2_ on Intron 1 hypomethylation, WT1 mRNA and lncRNA expression is related to HIF-1α stabilization and not due to off-target, nonspecific toxicity, including the observations that these effects are not mimicked by doxorubicin (Figs. 2B and D) and that they are dependent on HIF-1α stabilization (Figs. 5 and 6). There is an extensive and growing literature discussing hypoxia-mediated changes in histone methylation [27], but little is known about the effect of hypoxia on DNA methylation. Although there have been reports of global changes in DNA methylation related to hypoxia through inhibition of DNMT expression [28, 29], exposure of animals to chronic hypoxia has been linked to increases in DNA methylation [30], making the effect of oxygen tension on DNA methylation very unclear. This is the first example of a gene whose expression is regulated by a hypoxia-mediated change in DNA methylation, and our results support a model wherein hypoxia reduces both expression and activity of DNMT and increases expression of TET DNA demethylases, resulting in decreased methylation of CpG islands and increased gene expression.

**Fig 9:**
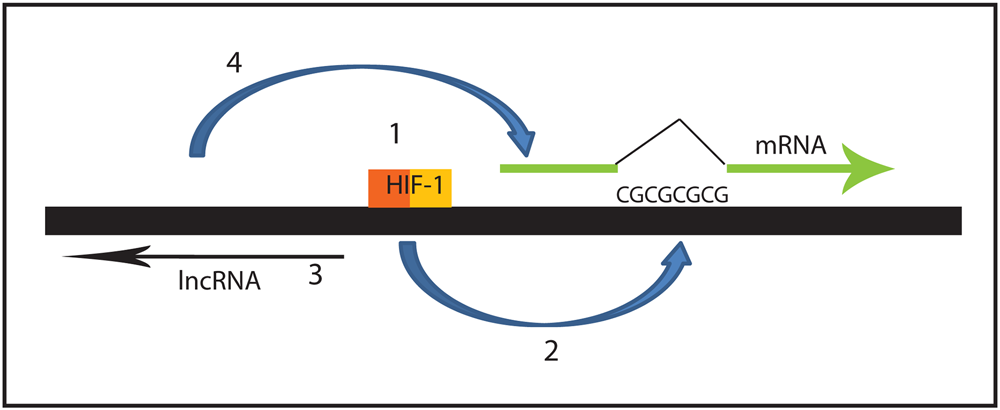
Model of WT1 mRNA regulation by hypoxia. This schematic diagram shows HIF-1 binding to the HRE in the WT1 promoter (1; requirement of HIF-1α demonstrated in Fig. 4), which leads to demethylation of the CpG island in Intron 1 (2; role of demethylation demonstrated in Fig. 1), resulting in expression of the lncRNA (3; expression shown in Fig. 3), which induces mRNA expression (4; requirement of lncRNA for mRNA expression is shown in Fig. 6).

Our studies also uncovered a novel mechanism by which hypoxia-mediated hypomethylation of a CpG island can increase gene expression. We found that demethylation of the Intron 1 CpG island allows the transcription of an antisense-oriented lncRNA, and that expression of the lncRNA is necessary for WT1 mRNA expression (Fig. 9). Our work has revealed that this lncRNA plays a critical role in WT1 regulation under a variety of circumstances. Myeloid leukemia cell lines that express WT1 also express the lncRNA, while cell lines that do not express WT1 do not express the lncRNA. Although a correlation between expression of this lncRNA and WT1 mRNA was previously reported by Moorwood et al. in developing kidney [31], and has been reported in other cell types (including leukemia) as well [32], a causal relationship was hypothesized in these prior reports but was not demonstrated. Importantly, our current work is the first direct demonstration of a causal relationship between the antisense-oriented WT1 lncRNA and WT1 mRNA expression. Silencing the lncRNA with shRNA blocks WT1 expression in K562 cells as well as blocking induction of WT1 by hypoxia in U937 cells. In addition, our work is the first demonstration that hypoxia regulates WT1 lncRNA expression, and is the first demonstration that the effect of hypoxia on WT1 mRNA expression is mediated through upregulation of the WT1 lncRNA. Thus, it appears that the WT1 lncRNA is a key regulator of both constitutive and hypoxia-regulated WT1 expression.

Taken together, our results support a novel model for hypoxia-mediated regulation of gene expression: hypoxia causes both a decrease in DNMT expression and activity, and an increase in TET2 and TET3 expression and activity, resulting in hypomethylation of a CpG island, which allows the expression of a lncRNA that is necessary for the expression of a protein-coding mRNA. Several questions remain unanswered regarding this model, and will be addressed in future work. Our work did not address the role of the HRE in regulating WT1 expression. The HRE is located in the 5’ WT1 promoter, within 200 bp of the lncRNA transcription start site. Our results clearly demonstrate that HIF-1α is necessary for hypoxia-mediated upregulation of both the lncRNA and the mRNA, but whether the HRE is necessary for lncRNA expression, and how HIF-1 binding to the HRE alters lncRNA expression and interacts with the lncRNA to increase WT1 mRNA expression remain to be determined. The mechanism by which lncRNA increases mRNA expression is also unclear. Although Dallosso et al suggested that lncRNA may stabilize mRNA in the cytoplasm [32], this was not proven. Were this mechanism to be true, expression of exogenous lncRNA, as from a lentiviral vector, would be expected to increase the level of mRNA seen. Our data suggest that lncRNA must be expressed in cis relative to the mRNA, since transduction of U937 cells with a lentiviral vector directing expression of the lncRNA did not upregulate WT1 mRNA expression. Thus, stabilization of the mRNA by the lncRNA is unlikely to be the mechanism by which lncRNA upregulates mRNA and protein expression. In other systems, lncRNAs have been shown to affect gene expression by altering chromatin structure, especially histone methylation. We found hypoxia-sensitive changes in histone methylation in the WT1 locus, and showed that these changes are dependent upon expression of the lncRNA. Future work will determine the precise mechanism by which the lncRNA mediates changes in chromatin structure that ultimately modulate WT1 mRNA expression.

The influence of hypoxia on epigenetic regulation of gene expression is the subject of an intense research effort, and our results indicate a novel mechanism by which hypoxia can control DNA methylation—through the regulation of TET expression and activity. TET genes are increasingly recognized as key regulators of DNA methylation, and have been implicated in stem cell function, developmental biology, and cancer. Little is known about the regulation of TET expression, however. Our results point to hypoxia as a key regulator of TET and provide a solid link between oxygenation and epigenetic regulation of gene expression. Interestingly, inspection of 3 kb of the 5’ regions of the TET2 and TET3 genes revealed 6 potential hypoxia response elements, supporting our findings that expression of these genes is hypoxia-sensitive.

This link may be particularly important for hematopoietic stem cell biology. There is increasing evidence that hematopoietic stem cells reside in a hypoxic niche in the bone marrow [33]. WT1 is expressed in hematopoietic stem cells but not in committed progenitors [20], and has been postulated to play an important role in regulating stem cell quiescence and differentiation [19]. Our findings suggest a mechanism by which hypoxia can directly affect proliferation and differentiation of hematopoietic stem/progenitor cells—by regulating WT1 expression. Our findings support the following model: quiescent hematopoietic stem cells express WT1 because they reside in a hypoxic niche, and WT1 promotes this quiescence; as cells move (actively or otherwise) into regions of the marrow with higher oxygen tension, WT1 expression diminishes and the cells become more proliferative and begin to differentiate. Because the hypoxia-mediated upregulation of WT1 is dependent upon a lncRNA, it is likely that other genes are coordinately regulated with WT1. Future work will directly test this model, including identification of coordinately regulated genes that mediate the proliferation and differentiation of hematopoietic stem cells.

Finally, the role of TET proteins in stem cell biology and malignancy is an area of intense interest. Both TET1 and TET2 physically associate with NANOG and promote somatic cell reprogramming and the establishment of pluripotency [34]. TET1 and TET2 appear to play distinct roles in genomic imprinting, with TET2 being required for efficient reprogramming of embryonic germ cells and TET1 being important for erasing imprinting of Imprinted Control Regions through oxidation of 5mC [34]. TET2 appears to be specifically important for hematopoietic stem cell homeostasis, with deletion of TET2 causing increased self-renewal and a survival advantage compared with wild type HSC in competitive repopulation assays in mice [35]. Our findings that hypoxia can regulate expression of all 3 TET genes, at least in U937 cells, suggest potential molecular mechanisms to explain previously established links between hypoxia and hematopoietic stem cell function and between changes in DNA methylation and hematopoietic stem cell function [36, 37] that warrant further exploration in future work.

## Materials and Methods

### Cell Culture

K562, U937 and HL60 cells were cultured in RPMI 1640 medium (Invitrogen, Grand Island, NY) supplemented with 10% fetal bovine serum (Gemini Bio-products, West Sacramento, CA). 293T cells were propagated in DMEM (Invitrogen) supplemented with 10% fetal bovine serum. Cells stably transduced with shRNAs (Origene, Rockville, MD) were cultured in 0.5 mg/ml G418 (Mediatech Inc., Manassas, VA), or 2 μg/ml puromycin (Invitrogen) as appropriate. Cells were grown at 37°C in atmospheric O2 (21%) or in a sealed incubator chamber (Billups-Rothenberg, Del Mar, CA) in 5% CO2, 1% O2, and 94% N2 (Airgas East, Linthicum Heights, MD). Primary patient leukemia samples were obtained from our institutional leukemia bank. These samples are from diagnostic bone marrow aspirations. Collection and storage of these samples was performed under a protocol approved by the Johns Hopkins Institutional Review Board.

### Transfections

Cells were transfected using Lipofectamine 2000 (Invitrogen). For stable transduction of shRNA, 293T cells were grown to 80–90% confluence and then co-transfected with 3.0 μg of the shRNA plasmid (Origene) and 1.5 μg of pCL-Ampho packaging vector. Forty-eight hours after transfection, viral supernatants were collected. Cells were transduced by adding 2 ml of the viral supernatant along with polybrene to a final concentration of 4 μg/ml.

### Semiquantitative RT-PCR

Total RNA was harvested from cells using an RNeasy kit (Qiagen, Valencia, CA), and 1 μg of DNase I-treated RNA was reverse transcribed using iScript reverse transcriptase (Bio-Rad, Hercules, CA) and analyzed by PCR (see Table 1 for primer sequences).

### Quantitative RT-PCR

Primers were obtained from SuperArray Bioscience (Fredrick, MD). Quantification of gene expression was performed using a Bio-Rad MyiQ single color real time PCR detection system with SYBR Green chemistry. Quantification of gene expression was performed by the ΔΔCt method.

### Chromatin Immunoprecipitation

Chromatin immunoprecipitation (ChIP) was performed using a kit (Active Motif, Carlsbad, CA).

### DNMT1 assay

Nuclear extracts were prepared with a nuclear extraction kit (Active Motif) and assayed for DNMT activity using a colorimetric DNMT Activity/Inhibition assay kit (Active Motif).

### 5-hydroxymethylcytosine analysis

5-hydroxymethylcytosine assays were performed with the EpiMark 5-hmC and 5-mC analysis kit (NEB, Ipswitch, MA). Total cytosine, 5-methylcytosine (5mC), and 5-hydroxymethylcytosine (5hmC) were calculated using the following formulae:

%5hmC = [M2*(C1/C2)-M1]/C1

%5mC = [H1-M2*(C1/C2)]/C1

%C = (C1-H1)/C1

C1 is the Ct of the sample with genomic DNA only, C2 is the Ct of the sample with genomic DNA treated with T4-BGT, M1 and M2 are the Cts of the samples digested with MspI without and with T4-BGT respectively, and H1 is the Ct of the sample digested with HpaII without T4-BGT.

### Western Blotting

Total cellular protein was extracted with a QIAmp kit (Qiagen). Samples were run on 4–12% bis-tris gels in MOPS buffer followed by transfer onto polyvinylidene difluoride (PVDF) membranes and blocking overnight in TBS, 0.1% Tween-20, and 5% nonfat dry milk. WT1 primary antibody (Novus, Littleton, CO) was diluted 1:1000 into blocking solution, and the secondary antibody, mouse IgG (Invitrogen), was diluted 1:20,000. As a loading control, anti-Histone H3 (D1H2) antibody (Novus) was diluted 1:5000 in blocking buffer followed by a 1:20,000 dilution of HRP-conjugated rabbit IgG antibody (Invitrogen). Immunoblotting was followed by visualization with ECL Plus (GE Healthcare) on x-ray film.

### Bisulfite conversion and sequencing and Methylation-Specific PCR (MSP)

Genomic DNA was extracted with a Qiamp DNA mini kit (Qiagen). Bisulfite conversion was performed with an Epitect bisulfite conversion kit (Qiagen). For bisulfite sequencing, appropriate primers were used for PCR amplification of the bisulfite-converted gDNA followed by cloning into the pCRII-TOPO TA vector using a TA cloning kit (Invitrogen). Ten individual clones were sequenced on an Applied Biosystems 3730xl DNA Analyzer. For MSP, primers that specifically amplify methylated and unmethylated sequences of bisulfite-converted gDNA were used on the bisulfite-converted gDNA.

### WT1 lncRNA isoform cloning and sequencing

Semiquantitative PCR was performed using primers specific to WT1 lncRNA. Products were separated by electrophoresis and the two individual bands were isolated and purified using a QIAquick gel extraction kit (Qiagen), cloned into the pCRII-TOPO TA vector using a TA cloning kit (Invitrogen), and sequenced on an Applied Biosystems 3730xl DNA Analyzer.

### Statistical analysis

Experiments were performed in triplicate a minimum of three times. Results are expressed as mean ± SEM. Statistical comparisons were made using an unpaired two-tailed Student t test with Prism v5.0 software (GraphPad Software, Inc., La Jolla, CA). A p value < 0.05 was considered significant.
